# Can Probiotics and Diet Promote Beneficial Immune Modulation and Purine Control in Coronavirus Infection?

**DOI:** 10.3390/nu12061737

**Published:** 2020-06-10

**Authors:** Ana H. A. Morais, Thais S. Passos, Bruna L. L. Maciel, Juliana K. da Silva-Maia

**Affiliations:** 1 Nutrition Postgraduate Program, Center for Health Sciences, Federal University of Rio Grande do Norte, Natal, RN 59078-970, Brazil; lima_bruna@yahoo.com.br (B.L.L.M.); juks3107@gmail.com (J.K.d.S.-M.); 2 Biochemistry Postgraduate Program, Biosciences Center, Federal University of Rio Grande do Norte, Natal, RN 59078-970, Brazil; 3 Department of Nutrition, Center for Health Sciences, Federal University of Rio Grande do Norte, Natal, RN 59078-970, Brazil; thais_spassos@yahoo.com.br

**Keywords:** *Lactobacillus gasseri*, COVID-19, SARS-CoV-2, viral infection, purine, immune system, nutritional intervention.

## Abstract

Infection caused by the SARS-CoV-2 coronavirus worldwide has led the World Health Organization to declare a COVID-19 pandemic. Because there is no cure or treatment for this virus, it is emergingly urgent to find effective and validated methods to prevent and treat COVID-19 infection. In this context, alternatives related to nutritional therapy might help to control the infection. This narrative review proposes the importance and role of probiotics and diet as adjunct alternatives among the therapies available for the treatment of this new coronavirus. This review discusses the relationship between intestinal purine metabolism and the use of *Lactobacillus gasseri* and low-purine diets, particularly in individuals with hyperuricemia, as adjuvant nutritional therapies to improve the immune system and weaken viral replication, assisting in the treatment of COVID-19. These might be promising alternatives, in addition to many others that involve adequate intake of vitamins, minerals and bioactive compounds from food.

## 1. Introduction

COVID-19, the disease caused by the new coronavirus SARS-CoV-2, was officially declared a pandemic by the World Health Organization on 11 March 2020 [1]. Coronaviruses (CoVs) belong to the genus coronavirus and the family Coronaviridae. All CoVs are pleomorphic, single-stranded RNA viruses, positive sense, polyadenylated, containing crown shape peplomers with a size of 80–160 nM and 27–32 kb [2]. 

The initial analysis of the new coronavirus genome showed a typical genomic structure belonging to the β-coronavirus cluster, including SARS-CoV and MERS-CoV [3]. According to Chen et al. [3], CoV recombination rates are very high due to the constant development of RNA dependent DNA polymerase (RpDd) transcription errors, and this has been one of the main targets for the discovery of drugs [4]. These mutations facilitate recognition in the human cell receptors by SARS-CoV-2 [5]. As is the case with other infections, SARS-CoV-2 infection presents specific outcomes, and one of the facts contributing to these variations is SARS-CoV-2’s high mutation rate [6,7].

Coronaviruses (CoVs) are a group of viruses responsible for a broad spectrum of diseases in several species. Human coronaviruses cause various respiratory diseases, some like the common cold, and others more severe, such as pneumonia, bronchitis, severe acute respiratory syndrome, and Middle Eastern respiratory syndrome [7]. In this sense, the infection can range from asymptomatic to oligo symptomatic to a clinical manifestation of the disease, causing symptoms mainly in the respiratory tract, which might lead to intensive care. It might also affect the gastrointestinal, hepatic and neurological systems [6,7,8]. 

Calculations based on World Health Organization data from Wuhan in China indicate that 14% of infected cases are severe, 5% require intensive care and 4% die [9,10]. The Chinese population was the first affected by the unprecedented outbreak caused by the new coronavirus. However, despite rigorous global efforts to contain the virus through social isolation measures, COVID-19’s incidence keeps increasing, and, currently, affects hundreds of countries worldwide [11].

There is still much to investigate, especially regarding prevalence and mortality estimates, since the real dimension of present and future COVID-19 consequences is not yet well established [12,13]. Nevertheless, the association of COVID-19’s higher mortality with senescence, preexisting diseases, and availability of health care services is well-accepted [14,15,16], and could be different in distinct countries affected by this pandemic. Unfortunately, all determinants of SARS-CoV-2 infection evolution, either asymptomatic or to clinical disease, remain unclear. Due to this easy infection transmissibility, the scientific community has joined efforts to combat COVID-19.

Currently, there are no vaccines or treatments registered for COVID-19. However, several studies have been published which highlight the alternatives related to the general treatment for viral infections [17,18,19,20,21]. Zhang and Liu [22] have added nutritional interventions, showing the virus target and related functions. Among these nutrients are A, C, D, E, and B complex vitamins, especially B2, B3, and B6. The effects of these vitamins, although not consensual for vitamin E [23], are known and related to the improvement of the immune response to viral infections treatment, from in vitro, preclinical, and clinical studies [24,25,26,27,28]. 

Considering minerals, Zhang and Liu [22] discuss adequate consumption of zinc, selenium, and iron. These minerals can have effects not only on the symptoms related to coronaviruses, such as diarrhea and lower respiratory tract infection but also on COVID-19 itself, with a study demonstrating some mechanisms of action [29,30,31]. Intracellular high levels of Zn^2+^ can compromise the RNA replication of several viruses that interfere in viral polyprotein processing [29]. Te Velthuis et al. [29] have demonstrated that increased Zn^2+^ concentrations inhibited the replication of RNA-dependent RNA polymerase (RdRp) from SARS-CoV nsp12. Omega-3 polyunsaturated fatty acids were also emphasized [32]. Another nutritional intervention reported was the administration of prebiotics and probiotics, since imbalance in the intestinal microbiota was observed in patients infected with COVID-19 [33]. 

Recently, the European Society for Clinical Nutrition and Metabolism (ESPEN) published a document providing concise guidance for the nutritional management of COVID-19 with ten practical recommendations [34]. However, this guideline is directed to patients in intensive care units (ICU), with morbidities and older age. The American Society for Parenteral and Enteral Nutrition (ASPEN) has also published some recommendations regarding mainly hydration, caloric and protein intake for patients with COVID-19 requiring ICU [35]. Although these nutritional therapies are based on scientific evidence, the existing guidelines are still limited to critical patients with COVID-19 [36]. 

Thus, nutritional recommendations to prevent the evolution of new coronavirus infection and the nutritional status of non-hospitalized patients has not been appropriately considered in the treatment of COVID-19 at this time, as with other viral infectious diseases. Some studies have shown the importance of early nutritional supplementation for non-critical patients hospitalized with COVID-19, emphasizing the importance of a balanced diet, and the necessity of unprocessed and healthy food choices [37,38,39,40]. Nonetheless, measures of primary care to avoid the aggravation of COVID-19 are barely mentioned despite all the attention given to combatting this pandemic combat by United Nations agencies [41]. 

The interaction between nutrition and infections is well accepted and valued by generations of health-care professionals. Before the use of antibiotics, diet was an integral and essential part of infection management. This strategy needs to be rescued, because understanding of the immune response and nutrition has considerably expanded. However, few studies have assessed the impact of nutritional interventions in the management of infectious diseases, and most observations of the interaction between nutrition and infections come from epidemiological studies [42].

The global pattern of food consumption has been associated with increased inflammation and uncontrolled infectious processes related to lower immune system responsiveness [43,44,45,46,47]. Additionally, the impact of the consumption of ultra-processed foods has been related to imbalances in the human intestinal microbiota [48]. Dysbiosis is closely associated with changes in the dynamics of the immune system [49].

Probiotics can help maintain or restore the balance of the intestinal microbiome and, consequently, improve the immune system’s response against aggression to the human organism [50]. Among the mechanisms of probiotics that corroborate with intestinal and systemic health is the competition with pathogenic microorganisms for adhesion sites and nutritional sources, the secretion of antimicrobial peptides, the action of metabolites, nucleosidase activity, and even immunomodulation by signaling pathways of intestinal and immune cells [51,52,53,54,55,56].

Besides the mechanisms well explored for probiotic action [51,52,53,54,55,56], a metabolic pathway has recently aroused the interest of researchers. This route involves the intestinal purine metabolism, possibly one of the explanations for the benefits achieved with the use of probiotics [57,58], to improve the immune system in cases of viral infections. Studies also show the association between high serum urates and the severity of HIV infection [59], which can help understand how the consumption of purine-rich foods could influence worsening clinical conditions in viral infections, such as those caused by a coronavirus. Thus, curiosity and interest in nutritional therapies to promote the reduction of purine intake have increased, whether using probiotics [33,52,53,57,58] or by dietary restriction advice on source foods [60], and consequently control of serum uric acid concentrations [61]. This behavior can positively influence the health of individuals with viral infections. 

Future research may specifically elucidate the effectiveness of adjuvants as part of COVID-19 treatment. This review adds the scientific data available so far, demonstrating the role of the microbiota in the immune response and the application of probiotics in coronavirus infections. Lactobacilli are lactic acid bacteria with probiotic potential. Several health benefits have been attributed to a specific strain of lactobacilli, *Lactobacillus gasseri* [62]. Studies have shown the action of *L. gasseri* in different viral infections, including respiratory [63,64]. We also review the importance of other nutritional interventions related to the consumption of food sources of purine. The findings may contribute to combating COVID-19 during this global health emergency.

## 2. Probiotics and Immunity

Researches have shown that microorganisms with the ability to modulate the intestinal and systemic immune response could be used in bacterial and viral respiratory infections to improve their outcomes [64,65,66,67,68,69]. 

The gastrointestinal tract (GT) presents a microbiome inhabited by a mass of active bacteria that are important for the maturation of immune cells, affecting human health status [51,70]. These bacteria belong mainly to three phyla: Bacteriodetes, Firmicutes, and Proteobacteria. However, the number and proportion of microorganisms differ according to the GT portion and individual characteristics [71,72]. 

An important factor that affects both gut microbiota and the immune system is diet. Data have shown that human gut microbiota in industrialized societies is very distinct from the recent ancestral microbiota of humans [48]. Ultra-processed foods modify the gut environment, being trigger factors for low-grade systemic inflammation and oxidative stress [73]. Recent work proposed the term “microbiota insufficiency syndrome”, and this could be linked to many non-communicable chronic diseases [74].

An unbalanced state of the microbiome, called dysbiosis, is characterized by overgrowth of pathobionts, loss of commensals, and lower diversity. Once established, it can disturb the local mucosal and systemic immune cells [49]. Given the association of dysbiosis with the etiology of several diseases, approaches, such as administration of probiotics that control pathogenic microorganism growth and modulate immune response may promote intestinal and systemic health [49,62]. 

Lactobacillus and Bifidobacteria are the most common microorganisms used as probiotics, but yeast *Saccharomyces boulardii* and Bacillus species are also widely applied [72]. Probiotic bacteria are mostly members of the gut microbiome and can be incorporated into food or administrated in isolated form. However, probiotic microorganisms are strain-specific, and not all bacteria have this property [51]. Firstly, they have to survive in acid conditions of the stomach to exhibit beneficial health impacts [75]. Then these microorganism strains, such as Lactobacillus, can maintain the ecological balance of the host intestinal microbiota by reinforcing intestinal flora and inhibiting harmful bacteria [70]. Several mechanisms of action can also affect the immune system. Improvements in immune response is a strategy to combat infections, bacterial, and viral infection [50,66,67,68].

Probiotic supplements with lactobacilli and bifidobacterial strains are well-established as part of the treatment of infectious diarrhea caused by rotavirus. Azagra-Boronat and Massot-Cladera [76] showed that probiotics decreased the severity and incidence of diarrhea in the preclinical assay. Furthermore, probiotics and their metabolites have been investigated as adjuvants in immunotherapy for viral hepatitis cirrhosis [77]. 

Secondary bacterial pneumonia is a significant complication during epidemic and pandemic viral respiratory infections that can increase morbidity and mortality. Virus infection promotes bacterial attachment and colonization, disruption of epithelial barriers, and alteration of the innate immune response in the respiratory tract. A study showed that peptidoglycan from immunobiotic *L. rhamnosus* CRL1505 improved respiratory antiviral innate immune response, and decreased bacterial transmigration across the lung and pulmonary inflammatory damage in infant mice [65]. 

A meta-analysis published by Cochrane [69], and other works, demonstrated the efficacy of probiotics in reducing the incidence and duration of acute respiratory tract infections of viral origin and the need for several antibiotic courses [78,79]. A study reported that generalized probiotic intake in the US population for 2017–2018 would have saved 373 million USD in health care for flu-like acute respiratory tract infection [80]. It is essential to avoid the overuse of antibiotics, which can lead to the development of antibiotic-resistant pathogens and alter the composition and functions of the microbiota, causing long-lasting harmful effects for the host [81]. Reasonable care should be applied in antibiotic drug utilization in upper respiratory tract acute infections, assessing severity, clinical signals, and complication risks. In this sense, probiotics could be an adjuvant in respiratory infectious disease treatment, optimizing antibiotic administration [69]. 

### 2.1. Probiotic Mechanisms of Action

Although the most widely discussed mechanism of probiotic action is the inhibition of the growth of pathogenic bacteria [51], other mechanisms explain the antagonistic effects of probiotics, among them adhesion and co-aggregation ability, competition for binding sites and nutritional sources, secretion of antimicrobial substances, enhancement of intestinal barrier function by regulation of tight junctions and mucins expression, along with immunomodulation by interaction to receptors of microorganism-associated molecular patterns [51,75,82,83,84]. 

The intestinal barrier is composed of a mucus layer, intestinal epithelium, and underlying lamina propria. The mucus layer is a physical barrier to avoid direct contact with the intestinal epithelium. Several specialized cell types are present in the intestinal epithelium: enterocytes responsible for absorption, Paneth cells specialized in synthesizing and secreting antimicrobial peptides, and mucus-secreting Goblet and entero-endocrine cells. The mucosal permeability is under the control of the tight junction proteins. The lamina propria consists of dendritic cells, macrophages, plasma cells, and B and T lymphocytes [50,85].

The effectivity of probiotic bacteria depends on their adhesion capacity to the host, to other bacterial cells (co-aggregation), or even to the same species (auto-aggregation). In this way, they can colonize and promote immunomodulatory effects, besides stimulating gut barrier and metabolic functions [82]. 

Short-chain fatty acids (SCFA) (butyrate, propionate, and acetate) are well-known products from microbiota fermentation with an immunoregulatory function due to the influence in the extracellular and intracellular signaling molecules. SCFA can bind to cell surface G protein-coupled receptors and modulate the immune function indirectly. These compounds can inhibit histone deacetylases, the cell interior, and regulate gene transcription in the immune response. Besides, they can promote B-cell differentiation and antibody synthesis, improving the antibody-antigen response [86]. Other metabolites from microbiota metabolism are essential amino acids, such as tryptophan, which are closely related to the immune system. Tryptophan metabolites attenuate TNF-α-induced activation of NF-κB and reduce expression of the proinflammatory chemokine, besides strengthening the intestinal epithelial barrier by fortifying tight junctions. The intestinal microbiota can also synthesize vitamins from complex B, and vitamins play a vital role in regulating the immune system [72]. 

Microbiota sensing, by epithelial cells, dendritic cells (DCs) and macrophages, is regulated by pattern recognition receptors (PRRs), which interact with microorganism-associated molecular patterns (MAMPs) driving the cascade of the immune response. Therefore, probiotics can modulate the immune system through the binding of their MAMPS (lipoteichoic acids, peptidoglycan, S-layer proteins, and nucleic acids) with PPRs (toll-like receptors, NOD-like receptors, C-type lectin receptors) expressed in the host intestinal mucosa [62,87]. Interestingly, variations in MAMP profiles have been attributed to differential immunomodulatory capacities of probiotic strains [87]. Human myeloid DC treated with Lactobacillus (either *L. gasseri*, *L. johnsonii* and *L. reuteri*) showed a three-fold induction of TLR-2 transcripts, and induced T cells toward T helper 1 polarization [88]. 

The response against food and commensal antigens is regulated by T lymphocytes as well as the immune response to combat pathogenic microorganisms [51]. Probiotic functions involve the action on T helper (Th) and regulatory T (TReg) cells in the lamina propria [87]. Depending on the stimulus, naive T cells can be differentiated toward a Th1, Th2, Th17, or Treg response according to the profile of secreted cytokines [89]. Probiotic bacteria can influence mucosal immune responses and T cell differentiation by the induction of different cytokines secretion [51]. This secretion is associated with the equilibrium in DC stimulation and tolerance promoted by the presentation to Lactobacillus cells that, consequently, is reflected in the balance of the Th1/Th2 responses, besides being important to maintain homeostasis in symbiotic bacteria [90]. 

The anti-inflammatory effect of a probiotic formulation (*L. rhamnosus*, *B. lactis*, and *B. longum*) was demonstrated. There was a significant increase in anti-inflammatory IL-10 production and decrease of proinflammatory cytokines IL-1β and IL-6 in the human macrophage cell line THP1 [91]. Moreover, treatment with probiotics shifted gut microbiota composition, increasing the ratio of Prevotella and Oscillibacter in mice. These bacteria produce anti-inflammatory metabolites and, consequently, decrease Th17 polarization, favoring the differentiation of anti-inflammatory Treg/Type 1 regulatory T cell in the gut of an experimental model [92]. Lactobacilli, including *L. gasseri*, activate human dendritic cells and lead toward a Type 1 T-helper polarization, as evidenced by higher secretion of IFN-γ and IL12. These cytokines act by switching naive or memory T cells towards Th1 responses, promoting immunity against infections and other diseases [88].

Probiotic bacteria can be adjuvants in vaccine outcomes, acting in T cell responses toward Th1 [88], enhancing B cell and antibody response, besides their protection against infections directly at gut mucosal level and through interactions with the innate immune system [93,94]. Lactic acid bacteria have been assessed as non-parenteral live mucosal vaccine vectors [95,96]. In addition, a clinical trial with 50 volunteers evidenced that oral intake of *L. fermentum* CECT5716 enhances the effects of influenza vaccination, increasing T-helper type 1 and the vaccine-specific IgA antibodies post-vaccination [97].

Immunoglobulin A (IgA) is part of the humoral response; this antibody is fundamental to adaptive immune response and is necessary to mucosal barrier function. IgA can bind to commensal and pathogen bacteria and toxins, blocking them, which can optimize the DC action and overall immune response. It has been demonstrated that probiotics can induce IgA-secreton, collaborating in maintaining immune surveillance [51,98]. Therefore, probiotics induce protective humoral and cellular immunity.

Probiotics also regulate the expression of multiple intestinal genes. Specific probiotics regulate the expression of tight junction proteins, collaborating to preserve the integrity of the GT epithelium. The Lactobacillus strains were protective against *Escherichia coli* infection. The epithelial cells treated with the probiotic maintained higher levels of ZO-1 and other tight junction protein expression than those infected with the pathogen alone [84,99,100]. 

Probiotic bacterial strains (*Lactobacilli*, *Bifidobacteria*, and *Streptococci*) have also been shown to regulate mucins expression, improving the properties of the mucus layer and indirectly supporting the gut immune system [84]. The preserved mucus layer avoids the adherence of pathogenic microorganisms. Probiotics can also regulate gene expression of enterocytes and dendritic cells [72]. Another mechanism of action is related to the stimuli of secretion of antimicrobial peptides (bacteriocin) by gut microbiota, protecting the host against infections. Bacteriocin defends the organism against many pathogenic bacteria, such as *Staphylococcus aureus*, *Escherichia coli*, *Listeria monocytogenes*, and Salmonella [50]. These antimicrobial peptides are released by Paneth cells in response to enteropathogenic bacteria stimulation, contributing to the innate immune response of the GT. These peptides, such as lactocillin and other thiopeptides, have been studied for the production of drug-like molecules to clinical application as antibiotics [101]. 

In addition to the actions mentioned on immunity, *Lactobacillus* can influence the nucleotide metabolites in the intestine. *L. fermentum* ONRIC b0185, *L. fermentum* ONRIC b0195 e *L. pentosus* ONRIC b0223, presented in cell, culture high nucleosidase activity converting nucleoside into purine bases [54]. The Lactobacillus DM9218 strain also acts on the purine nucleosides (inosine and guanosine) [55,56]. *L. gasseri* PA3 has demonstrated an ability to reduce purine in foods and beverages [57,58,61]. Purines are essential to viral RNA synthesis. Reducing purine availability might slow down virus replication, holding down viral infections. Thus, reducing purine availability may be an important pathway to be explored in the use of probiotics during viral infections, besides their immune-modulatory actions. 

Concerning the effect on respiratory infections, lactobacilli have been associated with beneficial action in other viral infections. Recently, the anti-HIV effect of lactobacilli was investigated in human cervicovaginal and tonsillar tissues ex vivo, and cell lines were infected with HIV-1. Extracellular vesicles released by *L. crispatus* BC3 and *L. gasseri* BC12 protected from HIV-1 infection, a result attributed to the presence of protein and metabolites in these vesicles [102]. 

Thus, probiotics can help maintain a balance between inflammatory responses to pathogens and natural intestinal homeostasis and function. Promotion of homeostasis in the gut microbiome by probiotics could benefit the immune system as a whole. Consequently, the immune response to infections can be favored, and this may apply to SARS-CoV-2. Specific roles of probiotics in the immune response against viral infections will be discussed, and *L. gasseri* has been specifically addressed for its effects on immunity and purine control. *L. gasseri’s* effect on purine metabolism might reduce viral replication, and this should be explored for Sars-CoV-2 infection as discussed below. 

### 2.2. Lactobacillus gasseri

*L. gasseri* is one of six species in the *L. acidophilus* complex. *L. gasseri* has been described as a multifunctional probiotic and one of the dominant species in the human intestine [62]. These microorganisms can be isolated from human feces, breast milk, urogenital tract, and oral cavity [64,103,104]. Several health benefits were attributed to this probiotic through antimicrobial activity and immunomodulation [62]. 

Recently, lactobacilli were isolated from human feces, and thirteen were identified as *L. gasseri* by 16 rDNA sequencing, showing the genetic diversity and different probiotic characteristics [105]. The resistance of *L. gasseri* to the human gastrointestinal tract under difficult conditions is a property that allows this microorganism to colonize the gut, improving the microbiome [103,106]. A recent study showed that *L. gasseri* PA3 modulated the diversity of the microbiota after in vitro fermentation, increased the relative abundance of Lactobacillus and Escherichia, and decreased Bacteroides and Phascolarctobacterium [70].

Dendritic cells incubated with *L. gasseri* and bacteria produced more IL-10 and there was a higher level of natural killer cells in the spleen of mice treated with this probiotic [107]. In humans, a combination of *L. gasseri* CECT5714 and *L. coryniformis* CECT5711 stimulates NK cells and IgA concentration [108]. 

A study with *L. gasseri* 4M13 isolated from infant feces showed higher inhibition of nitric oxide production than other strains and reduced inflammatory mediators (TNF-α, IL-6, IL-1β) in LPS-stimulated macrophages. These results suggest *L. gasseri’s* potential to modulate proinflammatory cytokines [109]. 

Regarding its role in viral infections, *L. gasseri* strains have been intensively studied for the past few years. Human respiratory syncytial virus, an enveloped negative-sense RNA virus, is potentially at high risk of becoming severely symptomatic in elderly and immunocompromised individuals. The oral administration of *L. gasseri* SBT2055 in mice reduced the virus titer and attenuated the proinflammatory cytokines in the lung, and interferon and interferon-stimulated genes were upregulated [64].

The evolution of other viral respiratory infections was prevented through *L. gasseri* SBT2055 treatment, including Influenza A and human respiratory syncytial virus infections [63,64]. There was lower virus titer in the lungs, inflammatory cell infiltration, and IL-6 production in mice with Influenza A treated with *L. gasseri*. The probiotic enhanced the host defense system before the virus infection, increasing the gene expression of Mx1 and Oas1a, critical for the antiviral responses by IFN activation and antiviral immunity, and for enhancement of IgA production [63]. A human trial showed that the probiotic *L. gasseri* SBT2055 boosted the immune responses in healthy vaccinated subjects that received a trivalent influenza vaccine. This *Lactobacillus* strain stimulated humoral immunity, and the total IgG and IgA levels in plasma and sIgA production in saliva were also higher in the probiotic-treated group [110].

Other work has demonstrated that oral administration of *L. gasseri* TMC0356 stimulated local and systemic immune responses protecting against influenza virus infection. Clinical symptom scores were improved, pulmonary virus titers decreased, and mRNA expression of interleukin (IL)-12, IL-15, and IL-21 in Peyer’s patch and the pulmonary mRNA expression of IFN-γ, TNF, IL-12a, IL-12rbl, IL-2rb and perforin-1 increased significantly [111]. Therefore, *L. gasseri* promoted the down-regulation of viral replication in respiratory infections through the induction of antiviral gene expression [63,64,111].

A study demonstrated that *L. gasseri* SBT2055 induces transforming growth factor-β (TGF-β) expression in dendritic cells, activates Toll-like receptor-2 (TLR2) signal to produce IgA, and increases the rate of IgA in Peyer’s patch and lamina propria of the mouse small intestine. This probiotic also upregulated the gene expression of retinaldehyde dehydrogenase 2 (RALDH2), an enzyme responsible for the conversion of retinol into retinoic acid. The retinoic acids derived from the dendritic cell can promote IgA secretion via a retinoic acid-dependent mechanism that induces the expression of gut-homing receptors on B cells. Therefore, *L. gasseri* could enhance host immune responses and protect against infections [112]. Furthermore, *L. gasseri* OLL2809 promoted an important modulation of several proteins secreted from immature dendritic cells, reinforcing the concept of a protective anti-inflammatory role attributed to this probiotic strain. *L. gasseri* OLL2809 not only led to modulation in classic immune mediators, but also influenced proteins involved in contractile and desmosome machinery, presenting a new mechanism by which it can modulate the immune response by dendritic cells [113].

Also related to anti-inflammatory activity, another study with distinct Lactobacillus strains found that only *L. gasseri* Kx110A1 mitigated the action of macrophages stimulated with lipoteichoic acid (LTA) and lipopolysaccharide (LPS), inhibiting the release of the proinflammatory cytokines TNF- alpha and IL-6 through a lower expression of the metalloptroteinase-17 (ADAM17) enzyme (TNF-alpha-converting enzyme). These findings show a new mechanism of *L. gasseri* in blocking proinflammatory cytokine production [114].

Therefore, *L. gasseri* can boost both the innate and adaptive immune responses. The mechanisms involved are a stimulus to the production of cytokines from dendritic cells and macrophages and IgA production from B cells, which are associated with Toll-like receptors (TLR)-2 signaling [112,115].

Furthermore, the promotion of homeostasis in the gut microbiome by probiotics could benefit the immune system as a whole. Data illustrate *L. gasseri’s* potential to contribute to the mucosal gut barrier, microbiome modulation, and human health in general [62]. A clinical study showed that *L. gasseri* CP2305 administration mitigated the decline of *Bifidobacterium* ssp. and the increase of Streptococcus ssp. promoted by stress in young adults, favoring a balanced microbiome [116]. This Lactobacillus has also promoted regulatory effects in the gut environment and function according to Sugawara et al. [117]. In this work, individuals treated with *L. gasseri* CP2305 showed higher *Bifidobacterium* content, lower *Clostridium* cluster IV content in feces samples, and fecal p-cresol, a putrefactive product, was significantly decreased in the CP2305 group. The reduced p-cresol in fecal content can suggest that *L. gasseri* altered the tyrosine metabolism and maintained a healthy intestinal environment, avoiding potentially toxic protein degradation products [117]. An experimental study in chickens has shown that probiotic interventions with *Lactobacillus* species combined with *L. gasseri* protected against influenza virus infection through microbiota modulation [118].

The hypothesis that *L. gasseri* can be potentially beneficial in viral infections has been supported by several studies, including clinical trials [64,110,111,115]. The data are not explicitly related to coronavirus infection. However, the research included viral infections such as influenza, a respiratory viral infection. Therefore, this information might suggest the actions of this lactobacillus strain in COVID-19, improving the innate and adaptive immune systems, and further studies should address this hypothesis. 

*L. gasseri* PA3 can reduce purines, nucleosides, nucleotides and nucleic acids [57,58], including inosine 5′-monophosphate (IMPDH2), inosine, hypoxanthine, guanosine 5′-monophosphate (GMP), and guanosine in rats [52,53]. These effects are important, as recently Gordon et al. [119] cloned, tagged and expressed 26 out of 29 SARS-CoV-2 proteins in human cells, to identify, by affinity purification coupled with mass spectrometry (AP-MS), human proteins that can interact with SARS-CoV-2 proteins. The study showed the host factors that mediate viral infection, helping to elucidate the effective molecular targets for therapies to combat SARS-CoV-2 infection. In the study, purine biosynthesis enzyme IMPDH2, which can be reduced by *L. gasseri*, could interact with the viral protein nsp14. *L. gasseri* PA-3 reduces the absorption of the main purines contained in food, and this property has been explored as adjuvant therapy in gout and hyperuricemia [61]. 

Several studies have revealed and detailed the structure and functions of SARS-CoV-2 polymerases, enabling the understanding and development of potential inhibitors [4,120]. These RNA viruses require RNA-dependent DNA polymerase (RdRPs) for various stages in their life cycle. For this reason, RdRPs are potential targets for drugs and other therapeutic interventions for treating diseases caused by this group of viruses [4,5]. RdRP is the central catalytic subunit in RNA synthesis machinery for CoVs. An RdRP domain catalyzes the replication and transcription of the ~ 30 kb CoV RNA genome in the C-terminal part of nsp12, one of 16 replicase subunits [2,121]. Therefore, this allows the recognition of alternative substrates blocking purine availability for viral polymerase, resulting in errors during viral RNA synthesis [17,122]. Given the high similarity of amino acids that constitute the RdRps of SARR-CoV and SARS-CoV-2, studies have shown nucleotide analogs that can potentially inhibit SARS-CoV-2 polymerase.

According to Maxmen [4], some of these broad-spectrum antiviral agents, with proven efficacy, have been used when considering their viral inhibiting activities. Most of these purine nucleoside analogs block the replication of various RNA and DNA viruses by inhibiting inosine monophosphate dehydrogenase (IMPDH), an essential enzyme involved in guanosine triphosphate (GTP) biosynthesis. Others also inhibit viral RdRP activity, acting like a zinc ionophone, blocking mRNA capping and inducing mutations in RNA-dependent viral replication [4,17,123]. However, clinical application is limited due to serious side effects associated with high doses [124,125,126]. 

The possibility of using several antiviral drugs in the treatment of COVID-19 emerges, such as Favipiravir (C_5_H_4_FN_3_O_2_) [122], Alovudine (C_10_H_13_FN_2_O_4_) [17], Remdesivir (C_27_H_35_N_6_O_8_P) [127]; Azidotimidine (C_10_H_13_N_5_O_4_) [17], Atazanavir (C_38_H_52_N_6_O_7_) [128], Ivermectin (C_48_H_74_O_14_) [129] and Chloroquine (C_18_H_26_ClN_3_) [21,130]. These agents are known to be effective against other RNA viruses, including Influenza, HIV, Ebola, MERS-CoV, SARS-CoV, and SARS-CoV 2 in in vitro, experimental and clinical studies. Interestingly, some of these drugs influence the purine pathways [17,122,127].

Ahn et al. [131] proposed that SARS-CoV nsp12 can use both primer-dependent and primer-independent RNA synthesis activities, concluding that purine nucleotides are preferably utilized by nsp12 to restart RNA synthesis. Furthermore, the poly (A) tail at the 3ʹ end of the viral genome plays a regulatory role in the initiation during the minus-strand RNA synthesis. Finally, nsp12 needs a small region with less than 40 nucleotides at the ends of the viral genome and its complementary minus-strand RNA to initiate RNA synthesis. These results guide further modifications of these purine nucleoside analogs to generate broader and more potent anti-coronavirus agents.

## 3. The Role of Low-Purine Diets

Biological purines are small molecules found inside and outside cells. They consist of organic, aromatic, and heterocyclic compounds formed by a pyrimidine ring attached to an imidazole ring [132]. Purine bases are present in all plant and animal cells, are essential for life, as components of cellular energy systems (ATP and NAD), signaling (GTP, cAMP, and cGMP), and are associated with pyrimidines form RNA and DNA (adenine and guanine) [133]. There are two synthesis pathways for purine nucleotides: De Novo synthesis, and the salvage pathway. The first is the main synthesis pathway and the second is essential for brain and bone marrow [134].

Foods of animal and vegetable origin contain different amounts of purines, that when degraded generate uric acid, of which approximately 80% of excretion occurs through the kidneys [135]. Uric acid is considered an antioxidant due to the ability of urates to scavenge free radicals. However, the consumption of diets rich in purine can cause the overproduction of uric acid, which has low solubility in biological fluids, leading to the accumulation of serum urate in the blood above the saturation level (hyperuricemia). This clinical condition is responsible for causing health problems such as gout and is a risk factor for cardiovascular, renal, and metabolic syndrome [134,135,136].

Additionally, the main disorder related to purine metabolism is characterized by a deficiency of the hypoxanthine-guanine enzyme phosphoribosyl transferase (HPRT). This clinical condition is associated with the overproduction of uric acid, which can impair the hematological, immune, renal, and neurological systems. Thus, the genes associated with purine metabolism must be related to the metabolism of drugs [137].

When infected by viruses, human cells increase the demand for purine nucleotides, which are necessary for the synthesis of RNA and viral DNA. As a result, viruses obtain purine nucleotides through the action of the enzyme Inosine Monophosphate Dehydrogenase (IMPDH), which is essential for viral cell growth and differentiation. Therefore, IMPDH also becomes a target for antiviral pharmacological treatment [4,132].

According to studies, patients with HIV, for example, have an incidence of hyperuricemia higher than in the population of healthy individuals. This increase occurs because uncontrolled virus replication increases serum urate concentrations in response to increased cell turnover [59]. Carlucci et al. [138] concluded that the HIV-1 virus strongly influences the metabolism of the purine nucleotides in host cells. Considering these data documented for HIV, it is likely that the same preference for purine nucleotides may occur during the growth and differentiation of SARS-COV-2 cells. 

Thus, COVID-19 may be worsened in patients who ingest foods with high purine content, promoting an increase in uric acid concentrations. Probably, people with hyperuricemia or gout can be considered a risk group in cases of COVID-19. This proposition is reinforced by Ahn et al. [131], who suggested that coronaviruses use purine nucleotides to promote RNA synthesis. Lewandowski and Hsieh [139] mentioned rheumatologists’ concern about the increased risk of infection and death among patients with rheumatic disease, due to the increasing number of pre-admissions of COVID-19 patients with rheumatic disease. Due to the global urgency to answer this clinical question, the international rheumatology community created the COVID-19 Global Rheumatology Alliance to allow healthcare professionals from around the world to exchange information on case reports of patients with rheumatic disease diagnosed with COVID-19 to assist healthcare providers [140]. Currently, an anti-inflammatory widely used to treat patients with gout, colchicine [141], is undergoing clinical tests for the treatment of COVID-19. In addition to other drugs commonly prescribed by rheumatologists, hydroxychloroquine, glucocorticoids, intravenous immunoglobulin, anti-interleukin (IL) -1 and anti-IL-6 therapies, and Janus kinase inhibitors have also been tested [140].

Nutritional therapy is a promising adjunct to drug therapy, helping to reduce the intake of purines and control the concentrations of uric acid in the blood. This strategy could improve the clinical condition of patients with hyperuricemia resulting from viral infections, such as in AIDS [59], and probably COVID-19. To reduce purines from the diet, it is of fundamental importance to reduce the consumption of food sources containing a high concentration of these organic compounds, such as alcoholic beverages, sweetbreads, anchovies, sardines, liver, kidneys, brains, herring, mackerel and scallops [60,142]. Controlled consumption of sources with moderate concentrations of purine, for example, beef, pork, poultry, fish and seafood, asparagus, cauliflower, spinach, mushrooms, peas, lentils, dried peas, beans, oats, bran wheat, and wheat germ could also be beneficial [132].

According to the review study by Wu et al. [60], the purine content in food can change due to the type of processing, cooking, and storage. Based on the literature, the authors stated that lower storage temperatures could reduce enzyme activity, slowing down the nucleotide degradation pathways, affecting the content of purine bases. Besides, during industrial processing, food washing processes can reduce the content of hypoxanthine and adenine by around 60%, due to the higher solubility of these molecules [60]. This information can help individuals with hyperuricemia diagnosis to understand that some technological processes also help to reduce the purine content present in food.

On the other hand, modifying eating habits is a great challenge. It is also observed in cases of hyperuricemia; some research has shown innovative alternatives, which consist of developing foods and beverages with low purine content, using enzymatic degradation [143,144]. In this context, Purine Nucleoside Phosphorylase (PNP) is a crucial enzyme related to the purine degradation process, because it catalyzes the reversible phosphorolysis of purine nucleosides to the respective bases and sugars (ribose or deoxyribose) 1-phosphate. PNP acts on the salvage pathway allowing cells to reuse purine bases for nucleotide synthesis [145]. Therefore, this enzyme regulates nucleoside concentrations within living cells and is, therefore, the target of several types of pharmacological treatments, such as gene therapy for inherited immune deficiencies and solid tumors [146]. Although this idea is promising to help patients with hyperuricemia, Jankowska et al. [135] reinforced the idea that to be feasible it is necessary to overcome the technological challenge, which consists of producing enzymes in a way that preserves activity in the food and allows storage for long periods.

The use of probiotics as an alternative therapy to the use of drugs in order to treat hyperuricemia was discussed in the review by Prasad et al. [147]. They pointed out that, because pharmacological treatments for hyperuricemia have several side effects, researches have sought therapeutic alternatives less aggressive to the human body and effective in promoting the reduction of uric acid levels. In this context, research has involved the manipulation of the intestinal microbiome using probiotics. Therefore, probiotics can influence the metabolism of purines, breaking down the products inosine and guanosine to directly influence the reduction of uric acid levels. Also, Li et al. [56] investigated a strain of probiotics isolated from Chinese sauerkraut (DM9218-A) intra-gastrically administered in a rat model with diet-induced hyperuricemia. They observed that serum uric acid levels in animals were significantly reduced, suggesting that DM9218-A may be a promising candidate as an adjunctive treatment in patients with hyperuricemia during the initial period, besides its potential as probiotic acting in the prevention of hyperuricemia in the population. So, the use of probiotics is an interesting strategy, in cases of viral infections such as SARS-CoV-2, to reduce the purine nucleotides, and hinder the synthesis of RNA and viral DNA. 

Thus, this study highlights that one of the promising nutritional strategies to be included for SARS-COV-2 infection is the ingestion of probiotics, especially *L. gasseri*, which can have beneficial effects on the host’s health, activating the immune response in cases of infection. Besides, studies demonstrated that *L. gasseri* PA-3 promoted the absorption and incorporation of inosine and compounds related to purine metabolism in vitro and reduced intestinal purine absorption in clinical studies [52,53,57,58,61].

Studies on diverse aspects of COVID-19 are emerging now following the disease spread, such as a paper related to the treatment protocol established in China. The researchers have found that some patients with COVID-19 showed dysbiosis characterized by a lower level of *Lactobacillus* and *Bifidobacterium*. Thus, to regulate intestinal microbiota, the medical team included prebiotics or probiotics for these patients to reduce the risk of secondary infection due to bacterial translocation [33]. Future studies will elucidate the mechanisms and applications of probiotics in COVID-19 treatment. Nonetheless, there is already scientific data to support the action of probiotics enhancing the immune response in infection cases such as viral infections. So far, adjuvant therapies cannot substitute treatment with specific medication. However, the incorporation of adjuvant therapeutic factors in food could be a means of providing preventive medicine or better treatment.
